# Age-Associated Risk of Liver-Related Adverse Drug Reactions

**DOI:** 10.3389/fmed.2022.832557

**Published:** 2022-03-17

**Authors:** Yan-zhong Han, Yu-ming Guo, Peng Xiong, Fei-lin Ge, Jing Jing, Ming Niu, Xu Zhao, Zhao-fang Bai, Hai-bo Song, Xiao-he Xiao, Jia-bo Wang

**Affiliations:** ^1^College of Pharmacy, Chengdu University of Traditional Chinese Medicine, Chengdu, China; ^2^China Military Institute of Chinese Medicine, Fifth Medical Center of Chinese People's Liberation Army (PLA) General Hospital, Beijing, China; ^3^National Center for Adverse Drug Reaction Monitoring, Beijing, China; ^4^School of Traditional Chinese Medicine, Capital Medical University, Beijing, China

**Keywords:** adverse drug reaction, older adults, hepatotoxicity, pharmacovigilance, relative risk

## Abstract

**Objective:**

Aging population is generally considered more sensitive to adverse drug reactions (ADRs). Yet, big data-based quantitative evidence currently does not exist to support this concept. This study aims to investigate age-associated risks of liver-related ADR (L-ADR).

**Methods:**

Spontaneous reporting data from 2012 to 2016 were retrieved from the China National ADR Monitoring System. The risk ratio (RR) was used to quantify the relative risk of L-ADR of each age group. The reporting odds ratio (ROR) was used to quantify the correlation with the risk of L-ADR of each drug category or drug in older adults.

**Results:**

Totally, 64,702 L-ADR reports were retrieved, covering ages from 1 to 116, with a median age of 49. The RR values increased exponentially with the increase of age, which indicates that the relative risk of L-ADR increased by 33% for every 10-year increase in age. The age cutoff point for relative high risk of L-ADR was estimated at 52.0 years old (RR = 1). In 17 categories composed of 270 drugs, the top 3 drug categories with a high correlation to the risk of L-ADR in older adults were antiarrhythmic (ROR, 5.75; 95% CI: 4.45–7.42), antilipemic (ROR, 4.77; 95% CI: 4.53–5.02), and antihypertensive (ROR, 2.97; 95% CI: 2.59–3.41).

**Conclusions:**

This research illustrates quantitatively that aging is a potential risk factor for L-ADR, with a 33% increase in relative risk for every 10-year increase in age. Risk management should be addressed for older adults when those drugs with a high correlation to the risk of L-ADR are used.

## Introduction

The liver is the main organ of drug metabolism, so it is the most vulnerable to adverse drug reactions (ADRs) (1, 2). Liver-related ADR (L-ADR) is one of the most common severe ADR in clinical settings and may lead to liver failure and even death (3, 4). In the United States, about 50% of liver failure cases were caused by drugs (5, 6). L-ADR is also the leading cause of drug withdrawal from the market (7).

Aging is generally accompanied with a decrease in drug metabolism and elimination by the liver (8, 9). Thus, it can be considered that the incidence risk of L-ADR increases with age, which makes older adults at high risk for L-ADR (10, 11). However, this concept is largely based on the experience and studies on a smaller number of samples (12, 13). There is no consistent evidence that aging is a general risk factor for L-ADR, although it may be a risk factor due to certain medications, such as antimicrobials and cardiovascular drugs, being the most likely medications to cause DILI in older adults (14, 15). To date, there is no real-world big data-supported report on the age-associated risk of L-ADR that covers all drug categories.

With global aging, it is important to carry out pharmacovigilance for older adults to reduce the overall risk of ADR in the future and improve the overall healthcare quality throughout the world. However, there is a primary question of the age cutoff point in older adults experiencing L-ADR that needs to be addressed. To date, most of the pharmacovigilance research utilized the definition of older adults as ≥60 years initiated by the World Health Organization (WHO) (16–18), which was determined by the global population sociology. Concurrently, in new drug development and clinical trials, the Federal Drug Administration (FDA) defined ≥75 years as older adults, which was set according to the increase in life span and demographic changes in the USA (8). Nevertheless, these cutoff points for older adults may be not suitable for defining L-ADR risk cutoff age, since they are different discipline areas. In the internationally adopted consensus approach, the Roussel Uclaf Causality Assessment Method (RUCAM) is used for L-ADR, and the high-risk age cutoff point is set at ≥55 years (19, 20), which means a suspected L-ADR case will be added as one point for causality assessment. Notably, this age cutoff point in RUCAM is lower than those set by the WHO and FDA. What is more, it was derived mainly based on the opinion of experts at that time (8). Currently, there are few quantitative studies on real-world big data supporting the relationship between age and L-ADR risk.

With the largest population in the world, China has established a spontaneous ADR reporting and monitoring system that covers 1.4 billion people in all administrative regions and is managed according to the national unified standards, thus providing a unique source of real-world big data for investigating the age-associated risk of L-ADR. In this study, the influence of age on the risk of L-ADR and the age cutoff point for the high-risk population were explored using the widely adopted quantitative models, based on the L-ADR reports recorded in the Chinese National Adverse Drug Reactions Monitoring System (CADRMS) database. The quantitative relative risk of drug categories or a specific drug was also estimated to provide practical references for risk minimization in the management of L-ADR in older adults.

## Methods

### Data Description

This study was based on the information extracted from the Chinese National Adverse Drug Reactions Monitoring System (CADRMS) database accrued over a 5-year period between January 2012 and December 2016. The database specifically called for that each ADR report must be filed by patient's attending physician in a standardized format with the exact same set of information, including the patient's personal information (age, gender, date of birth, weight, height, address, and ethnicity); the information of the reporting physician including their medical specialty, affiliation; the patient's medical history, detailed information about the drug(s) used around the time when the ADR incident was noticed, including drug name, dose, dosing time, and latency time. Furthermore, the information is arranged in an access-friendly format that supports easy extraction of cases that are related to the liver as well as the data of interest through simple keyword-based searches.

### Study Design

This study is aimed at investigating the age-associated risk of L-ADR and the L-ADR risk of different drugs in older adults. Therefore, L-ADR cases were extracted from CADRMS based on the keywords (all in Chinese). To further guarantee the accuracy of the causality, the crude data were manually reviewed for final analysis on an independent case-by-case manner by 2 attending physicians in accordance with the adverse reaction casualty assessment of the World Health Organization-the Uppsala Monitoring Center (WHO-UMC) system.

Based on the L-ADR dataset, we calculated the risk ratio (RR) (21, 22) of L-ADR (Formula 1) of each age group. Then, regression analysis and modeling of the age-RR curve were used to explore the relationship between age and RR. Then, we used the cure to find the cutoff point of age between relative high-risk people and relative low-risk people which was defined as the cutoff point of age between older adults and non-older adults. Reporting odd ratio (ROR) (23, 24) (Formula 1) was used to quantify the correlation with the risk of L-ADR of different drugs in older adults. If RORs of drugs were >1, this means that these drugs have a high correlation with the risk of L-ADR in older adults; on the contrary, they have a low correlation.

Formula 1: $RR=\;\frac{a/b}{c/d}$

a, the reporting frequency (RF) for the target age group.

b, the number of people for the target age group nationwide.

c, the RF for the other age groups.

d, the number of people for the other age groups nationwide.

Formula 2: $ROR=\;\frac{e/f}{g/h}$

e, the RF of target drug category^*^ for older adults.

f, the RF of non-target drug categories for older adults.

g, the RF of target drug category for non-older adults.

h, the RF of non-target drug categories for non-older adults.

^*^, “drug category” could be either a drug subcategory or a specific drug.

The study was approved by the ethics committee of the Department of Liver Diseases of PLA General Hospital. The retrieved data did not contain personally identifiable information.

### Data Processing and Analyses

Since the CADRMS database is in Chinese, the keyword-based search was carried out with the Chinese translations of a set of terms that include “drug-induced liver injury,” “drug-induced liver damage,” “drug-induced liver disease,” “hepatotoxicity,” “toxic liver disease,” and “abnormal liver function.” At the same time, reports with keywords of non-drug etiology, such as “viral liver disease,” “alcoholic liver disease,” and “autoimmune liver disease,” were excluded. The search resulted in the recovery of 1,14,357 putative L-ADR cases.

**Figure 1:**
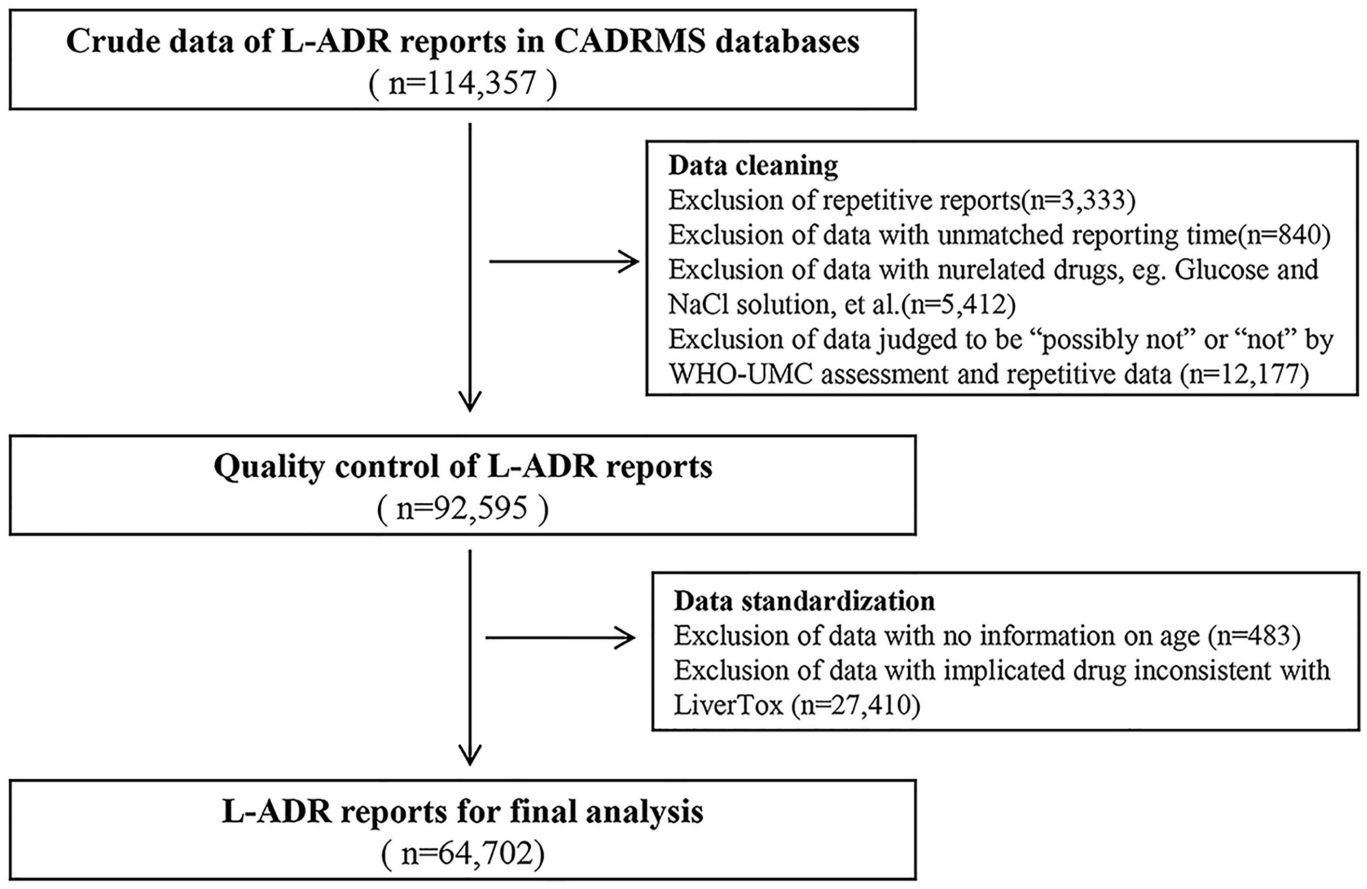
Data acquisition flowchart.

We performed CADRMS data cleaning as described (Figure 1). After being reviewed by 2 attending doctors in accordance with the WHO-UMC system, the cases that were judged as “possible,” “probable/likely,” or “certain” were screened out. Drugs implicated in the cases were classified based on a group of drugs that have been listed on the LiverTox (http://livertox.nih.gov) website (25). After data retrieval and review, a new database that contains 64,702 L-ADR-related cases (Figure 1) was constructed for the final analysis.

The cases in the new database were stratified by age group of 10 years (≤9, 10–19, 20–29, 30–39, 40–49, 50–59, 60–69, 70–79, 80–89, and ≥90 years old). The RR of different age groups was calculated separately in accordance with Formula 1. The population numbers of different age groups in China were referred to from the website of National Bureau of Statistics of China (http://www.stats.gov.cn/). Using regression analysis, we defined the age whose RR value is equal to 1 as the cutoff point of age between non-older adults and older adults. It meant that people older than the cutoff point were defined as older adults. Then, the ROR of different drug categories, subcategories, or specific drugs in older adults was calculated by Formula 2.

## Results

**Figure 2:**
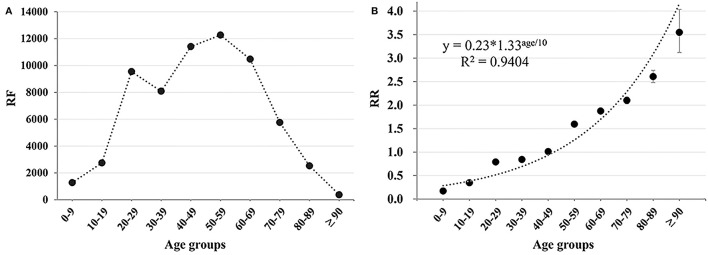
The age-associated trend of L-ADR. **(A)** The reporting frequency (RF) of L-ADR in each age groups; **(B)** the RR of different age groups. The regression equation of RR-age curve was RR = 0.23*1.33^age/10^.

The final dataset involved in the analysis contained 64,702 L-ADR-related cases, which included 270 drugs being classified in 17 drug categories and 40 subcategories (Table 1 and Supplementary Table 1) and covered ages from 1 to 116, with a median age of 49 (Figure 2A).

**Table 1:** ROR for different drug categories.

**Drug Categories**	**Cases (RF)**	**ROR**				
	** <52 years old (%), 35,940 (100)**	**≥52 years old (%), 28,762 (100)**	**Total (%), 64,702 (100)**	**Estimator**	**95%CI**	**Forest plot**
Antiarrhythmic	72 (0.20)	328 (1.14)	400 (0.62)	5.75	4.45–7.42	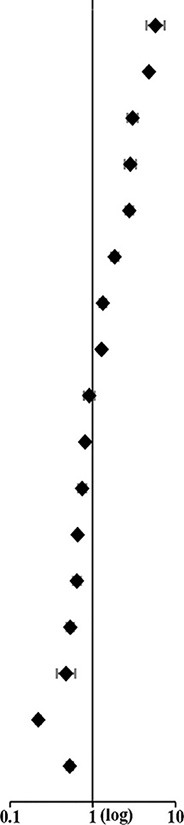
Antilipemic	2,194 (6.10)	6,806 (23.66)	9,000 (13.91)	4.77	4.53–5.02	
Antihypertensive	293 (0.82)	686 (2.39)	979 (1.51)	2.97	2.59–3.41	
Antidiabetic	234 (0.65)	525 (1.83)	759 (1.17)	2.84	2.43–3.31	
Antithrombotic	506 (1.41)	1,091 (3.79)	1,597 (2.47)	2.76	2.48–3.07	
Antifungal	643 (1.76)	936 (3.25)	1,579 (2.44)	1.85	1.67–2.04	
Antiulcer	803 (2.23)	847 (2.94)	1,650 (2.55)	1.33	1.20–1.46	
Antibacterial	3,012 (8.38)	3,006 (10.45)	6,018 (9.30)	1.28	1.21–1.35	
NSAID_S_	418 (1.16)	303 (1.05)	721 (1.11)	0.91	0.78–1.05	
Antineoplastic	4,391 (12.22)	2,904 (10.10)	7,295 (11.27)	0.81	0.77–0.85	
Antidepressant	816 (2.27)	489 (1.70)	1,305 (2.02)	0.74	0.66–0.83	
Antitubercular	13,255 (36.88)	7,983 (27.44)	21,238 (32.82)	0.66	0.64–0.68	
Antirheumatic	884 (2.46)	459 (1.60)	1,343 (2.08)	0.64	0.57–0.72	
Anticonvulsants	1,254 (3.49)	545 (1.89)	1,799 (2.78)	0.53	0.48–0.59	
Antiviral	207 (0.58)	79 (0.27)	286 (0.44)	0.48	0.37–0.62	
Antipsychotic	5,283 (14.70)	1,046 (3.64)	6,329 (9.78)	0.22	0.20–0.23	
Other^a^	1,679 (4.67)	725 (2.52)	2,404 (3.72)	0.53	0.48–0.58	

a *The drugs in the other category included acitretin, acyclovir, albendazole, alfuzosin, allopurinol, alpha interferon, alprazolam, androgen, baclofen, beta interferon, bosentan, bromocriptine, cetirizine, chlorzoxazone, cyproheptadine, deferasirox, deferoxamine, donepezil, entacapone, estrogens, febuxostat, flavonoid, lactobacillin, isotretinoin, mebendazole, memantine, methazolamide, methimazole, methoxsalen, montelukast, ondansetron, orlistat, propofol, propylthiouracil, rivastigmine, sevoflurane, sildenafil, tamsulosin, terbutaline, thyroxine, tibolone, tizanidine, and vitamin A*.

### Relationship Between Age and Relative Risk of L-ADR

In China, with the increase of age, L-ADR RF generally showed a trend of first increasing and then decreasing (Figure 2A). In contrast, the RR of age groups revealed increasing monotonously with age growth (Figure 2B). Notably, the age-RR curve was an exponential function and regression equation was RR = 0.23^*^1.33^age/10^ with a remarkably high regression coefficient (*R*^2^ = 0.9404, *p* = 0.000011). According to the regression equation, the risk of L-ADR increases by 33% for every 10-year increase in age. RRs for different age groups are shown in Supplementary Table 2.

Furthermore, the cutoff point of age between non-older adults and older adults was estimated by the regression equation as 52.0 years old (when RR = 1), which means that people older than 52 years may be high relative risk people to the L-ADR. Therefore, we defined people over 52 years old as older adults.

### ROR for Different Drug Categories

The RORs of different drug categories are presented in Table 1. Totally, eight drug categories whose RORs and 95% CI exceed 1.0 have a high correlation with the risk of L-ADR in older adults (≥52 years old), such as antiarrhythmic, antihypertensive, antilipemic, and antithrombotic. Besides, seven categories have a low correlation with the risk of L-ADR in older adults (≥52 years old), such as central nervous system (CNS) drugs (antipsychotic, antidepressant, and anticonvulsant) (Table 1). These categories that have a low correlation with the risk of L-ADR in older adults may be more likely to lead to L-ADR in non-older adults (<52 years old). The RORs of cardiovascular drugs, such as antiarrhythmics (ROR, 5.75; 95% CI: 4.45–7.42), antilipemic (ROR, 4.77; 95% CI: 4.53–5.02), and antihypertensive (ROR, 2.97; 95% CI: 2.59–3.41), occupy the top three in the risk ranking of drug categories, whereas antipsychotics (ROR, 0.22; 95% CI: 0.20–0.23) had the lowest correlation with the risk of L-ADR in older adults (Table 1).

### The Relationship Between ROR and RF

**Figure 3:**
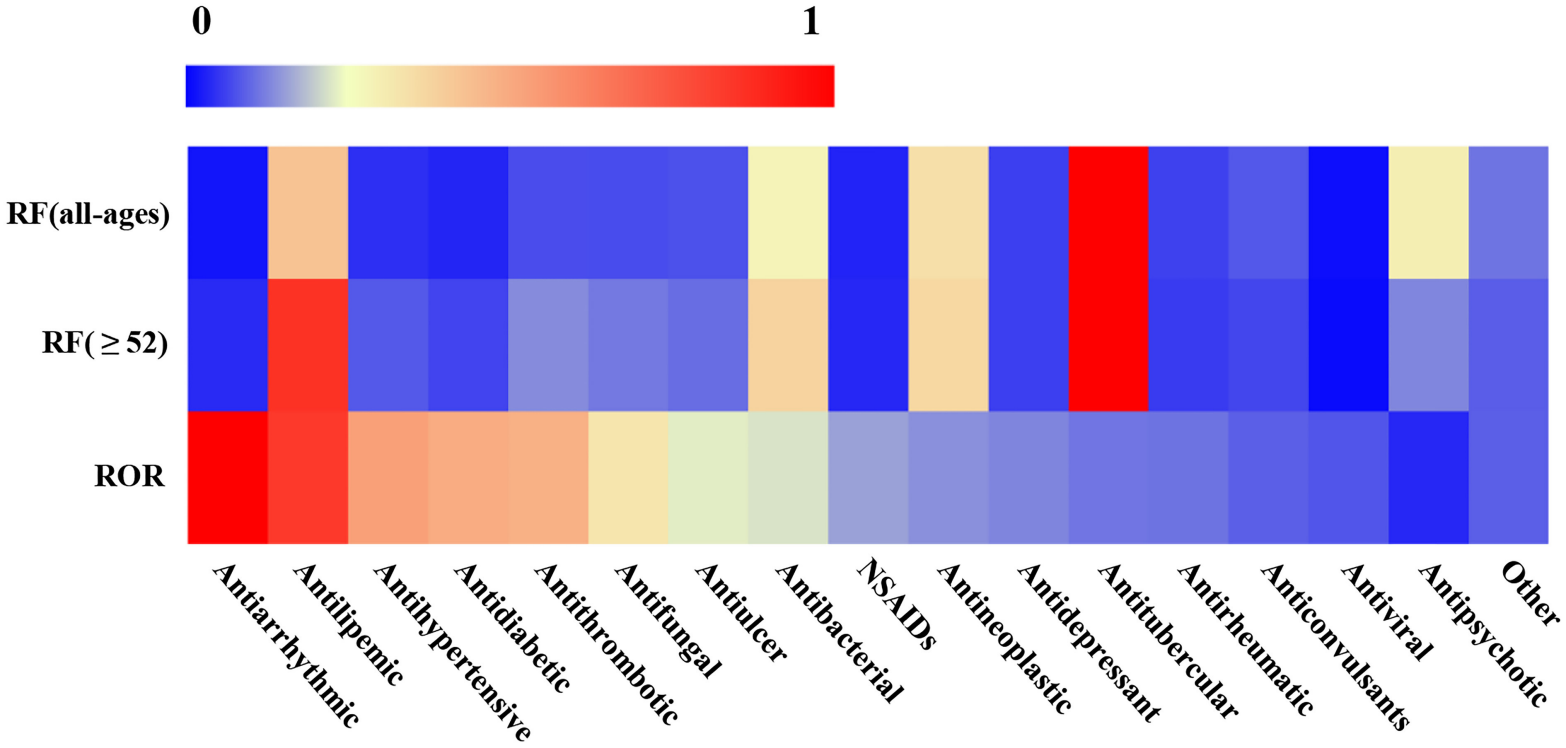
Heatmap of RF and ROR of different drug categories. The values of reporting frequency (RF) and ROR were normalized within 0–1, respectively, to make the comparability. The drug categories were ranked from left to right according to their ROR values except for other categories.

In addition, we noted that the RF of drug categories was not consistent with their ROR. For instance, the RF of antiarrhythmics was very low in all ages (RF= 400, 0.62%) or in older adults (RF = 328, 1.14%), but its ROR (5.75, 95% CI: 4.45–7.42) was very high. On the contrary, antitubercular drugs had very high RF in all ages (RF = 21238, 32.82%) or in older adults (RF = 7983, 27.44%), but its ROR (0.66, 95%CI: 0.64–0.68) was very low (Figure 3 and Table 1). The other four kinds of drug categories with high ROR, but low RF included antihypertensive, antidiabetic, antithrombotic, and antifungal drugs (Figure 3 and Table 1). This means that there is a limited connection between ROR and RF. RF cannot well and truly indicate the relative risk of L-ADR induced by drugs.

### ROR for Different Subcategories and Drugs

**Figure 4:**
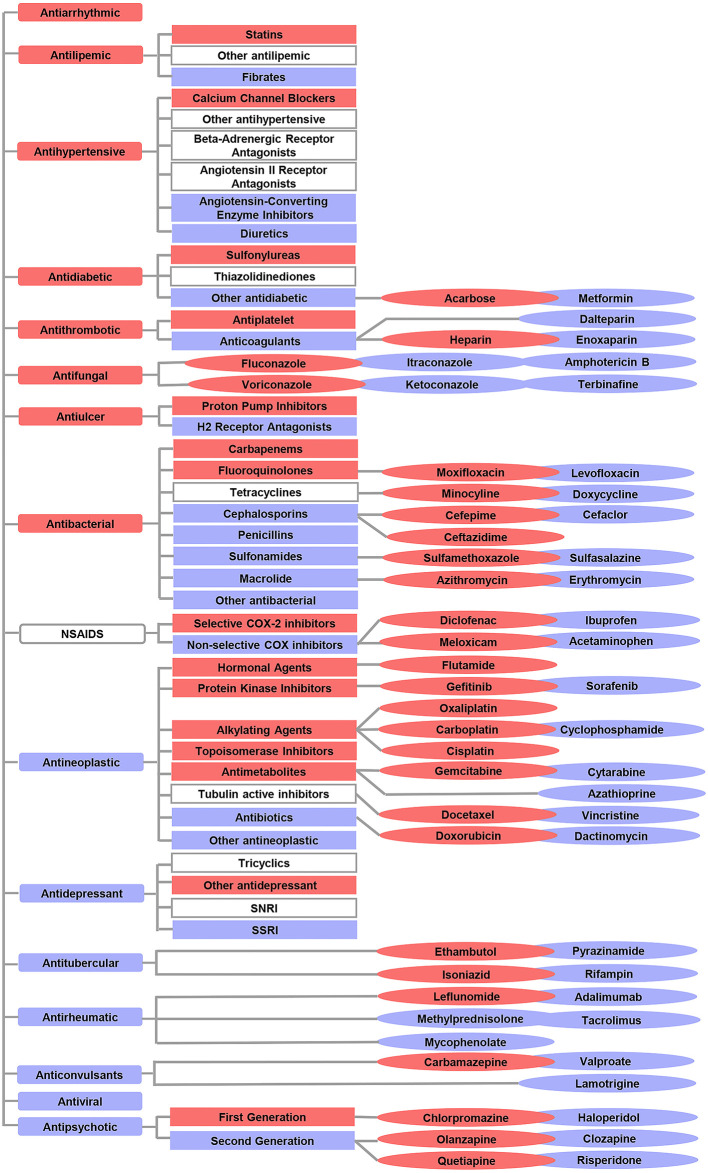
Overview of the differential risks of drugs with known hepatotoxicity. The red color indicates a high correlation with the risk of L-ADR in older adults, whereas the blue color indicates a low correlation with the risk of L-ADR in older adults. The symbols without color filling indicate drug categories or subcategories which were not identified with differential risks. 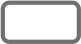 indicated drug categories; 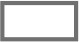 indicated subcategories; 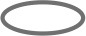 indicated drugs.

In addition to comparing the ROR of 17 drug categories, we also compared ROR values between subcategories or drugs with the same pharmacologic action. Overall, 15 subcategories and 27 drugs that have a high correlation with the risk of L-ADR in older adults were screened out, such as statins of antilipemic, sulfonylureas of antidiabetic, antiplatelet of antithrombotic, heparin of antiplatelet, leflunomide of antirheumatic, and so on. Risk profiles of L-ADR for all drugs are shown in Figure 4 and Supplementary Table 1.

## Discussion

In terms of overall drugs, RF of L-ADR increased first and then decreased with the increase of age. By contrast, the relative risk (RR) increased exponentially with a 33% increase in risk for every 10-year increase in age. This suggests that RF cannot accurately reflect the risk of L-ADR to a certain extent and that the relative risk of L-ADR in older adults is much higher than that in the younger population. This study is the first to verify the concept that “aging is a potential risk factor for L-ADR” from the overall drug. However, in terms of drug categories, aging is only a risk factor for certain drug categories, such as antiarrhythmic drugs, antihypertensive drugs, and antidiabetic drugs, whereas for CNS drugs, the relative risk of L-ADR in non-older adults is higher than that in older adults. This also confirms the correctness of some research results from the aspect of big data research (14, 26).

Globally, there is currently no research on the cutoff point of age in ADR of specific organs. In this study, 52 years was considered as the cutoff point between the low-risk age and high-risk age in L-ADR. Accordingly, 52 years was suggested as the age limit of older adults associated with L-ADR. Notably, it is nearly a decade earlier than the age limit of older adults (60 years old) in population sociology. However, many pharmacovigilance articles based on data mining chose 60 years old as the age limit of older adults (16–18), which may lead to underrepresentation of the older adults and deviation in the results of the articles. Thus, when conducting pharmacovigilance research related to older adults in the future, we should be first to calculate the age cutoff point between non-older adults and older adults based on different organ-specified ADR, such as liver, kidney, and heart, instead of simply referring to the age boundary of older adults in population sociology.

Moreover, this age (52 years) derived originally from ADR data analysis in the real world. Although it is slightly different from the DILI high-risk age boundary (55 years) in RUCAM, it supports its rationality to some extent. However, it also suggests that high-risk age for L-ADR or even DILI may be considered moving forward, which can be conducive to the development and improvement in ADR risk monitoring and drug safety alerts. Therefore, we propose to conduct research on the age distinction criteria of older adults associated with other organ-specified ADR to provide a reference for future pharmacovigilance research and cope with the global burden of ADR caused by aging.

Based on the age cutoff point of older adults derived from the above research, 15 drug subcategories and 27 varieties with a high correlation to the risk of L-ADR in older adults were screened out (Figure 4). This suggests that compared with other drugs of the same category, these 15 drug subclasses and 27 drug varieties are high-risk signals, leading to L-ADR in older adults. Therefore, from the perspective of drug safety, it may be necessary to be vigilant to them when selecting drugs for older adults. For example, in older adults with thrombotic diseases, it may be more reasonable to choose anticoagulant drugs with low correlation to the risk of L-ADR in older adults to replace antiplatelet drugs; among anticoagulant drugs, low molecular weight heparin, such as dalteparin and enoxaparin, may be safer for the older adults than heparin. In addition, existing data show that older adults with hypertension are at risk of heart failure, stroke, and even death. Diuretics and calcium channel blockers (CCBs) are the best choices when considering the therapeutic effects of hypertension (27, 28). Diuretics are also recommended as the first choice for the initial treatment of hypertension in older adults (29). This study also supports this view from the perspective of security. In addition, when the therapeutic effect of diuretics alone is not up to standard and needs to be combined with other antihypertensive drugs (angiotensin-converting enzyme inhibitors, angiotensin receptor blockers, or calcium channel blockers), we suggest that CCB should not be used as the first choice for combination therapy. Because this study found that CCB was a high correlation to the risk of L-ADR in older adults.

However, it should be noted that the drug signal with a high correlation to the risk of L-ADR in older adults generated by the quantitative signal detection method does not mean that there is an inevitable causal relationship between the drug and ADR. Whether this is a genuine safety issue requires a rigorous signal evaluation process that includes medical evaluation. The significance of mining for drug signals with a high correlation to the risk of L-ADR in older adults lies in that it can be used as a necessary reminder for liver function monitoring when these drugs are applied in clinical practice.

This study has limitations, as it is a retrospective study based on spontaneous ADR reports, which cannot give absolute values for age-related risk for a specific drug. A large-scale real-world prospective study is warranted in the future to get the incidence data.

In summary, based on real-world big data research, this study confirmed for the first time that aging is a potential risk factor for L-ADR at the overall drug level, and the cutoff point of age for the high L-ADR risk was estimated at 52 years. However, aging is only a risk factor for certain drug categories, such as cardiovascular drugs and hematological drugs. Not only did this study provide an age cutoff point reference for future pharmacovigilance studies related to L-ADR in older adults but also reference for future high-quality clinical and prospective studies, as well as provide multisource high-quality evidence for the prevention and control of L-ADR in older adults.

## Data Availability Statement

The original contributions presented in the study are included in the article/Supplementary Material, further inquiries can be directed to the corresponding author/s.

## Ethics Statement

The studies involving human participants were reviewed and approved by the Ethics Committee of the Department of Liver Diseases of PLA General Hospital. Written informed consent from the participants' legal guardian/next of kin was not required to participate in this study in accordance with the national legislation and the institutional requirements.

## Author Contributions

J-bW and Y-mG concepted and designed the study. Y-zH, PX, F-lG, JJ, MN, XZ, and Z-fB performed the trial and acquisition, analysis, and interpretation of data. J-bW and Y-zH drafted the manuscript. X-hX and H-bS contributed to critical revision of the manuscript for important intellectual content. All authors contributed to the article and approved the submitted version.

## Funding

This work was supported by the National Natural Science Foundation of China (nos. 82074112 and 81630100), National Science and Technology Major Project (no. 2015ZX09501-004-001-008), and the Project of China PLA General Hospital (nos. 2019-JQPY-003 and 2019MBD-023).

## Conflict of Interest

The authors declare that the research was conducted in the absence of any commercial or financial relationships that could be construed as a potential conflict of interest.

## Publisher's Note

All claims expressed in this article are solely those of the authors and do not necessarily represent those of their affiliated organizations, or those of the publisher, the editors and the reviewers. Any product that may be evaluated in this article, or claim that may be made by its manufacturer, is not guaranteed or endorsed by the publisher.
