# Supplementary material for: Age-Associated Risk of Liver-Related Adverse Drug Reactions
Source: Front Med (Lausanne). 2022 Mar 17;9:832557. doi: 10.3389/fmed.2022.832557 (PMC8968752; doi:10.3389/fmed.2022.832557)
Supplement: Supplementary file 1 [file Data_Sheet_1.docx]

**Supplementary materials**

**Supplementary Table 1 ROR of L-ADR for different drugs in older adults.**

| **Drug categories** | | **Cases (RF)** | | | **ROR** | |
| --- | --- | --- | --- | --- | --- | --- |
|  |  | **<52 years old** | **≥52 years old** | **Total** | **Ratio** | **95%CI** |
| **Antiarrhythmic** | | 72 | 328 | 400 | 5.75 | 4.45-7.42 |
|  | Propafenone | 2 | 14 | 16 | 1.56 | 0.37-3.54 |
|  | Amiodarone | 68 | 312 | 380 | 1.15 | 0.15-2.95 |
| **Antilipemic** | | 2194 | 6806 | 9000 | 4.78 | 4.54-5.03 |
| Statins | | 2058 | 6606 | 8664 | 2.18 | 1.75-2.73 |
|  | Rosuvastatin | 347 | 1218 | 1565 | 1.11 | 0.98-1.27 |
|  | Atorvastatin | 1193 | 3833 | 5026 | 1.00 | 0.91-1.11 |
|  | Simvastatin | 353 | 1087 | 1440 | 0.95 | 0.83-1.09 |
|  | Fluvastatin | 101 | 298 | 399 | 0.92 | 0.73-1.15 |
|  | Pravastatin | 48 | 139 | 187 | 0.90 | 0.65-1.25 |
|  | Lovastatin | 16 | 31 | 47 | 0.60 | 0.33-1.10 |
| Others | | 10 | 13 | 23 | 0.42 | 0.18-0.95 |
|  | Ezetimibe | 9 | 13 | 22 |  |  |
| Fibrates | | 126 | 187 | 313 | 0.46 | 0.37-0.58 |
|  | Fenofibrate | 120 | 184 | 304 | 4.02 | 0.99-16.38 |
|  | Gemfibrozil | 6 | 3 | 9 | 0.331 | 0.08-1.33 |
| **Antihypertensive** | | 293 | 686 | 979 | 2.64 | 2.63-3.48 |
| Calcium Channel Blockers | | 90 | 289 | 379 | 1.64 | 1.23-2.20 |
|  | Amlodipine | 46 | 173 | 219 | 1.43 | 0.89-2.30 |
|  | Diltiazemne | 2 | 8 | 10 | 1.25 | 0.26-6.01 |
|  | Nifedipine | 42 | 108 | 150 | 0.68 | 0.42-1.10 |
| Others | | 3 | 12 | 15 | 1.72 | 0.48-6.14 |
|  | Hydralazine | 2 | 9 | 11 | 1.50 | 0.10-23.07 |
| Beta-adrenergic receptor antagonists | | 57 | 125 | 182 | 0.92 | 0.65-1.31 |
|  | Metoprolol | 55 | 122 | 177 | 1.48 | 0.24-9.10 |
| Angiotensin II Receptor Antagonists | | 61 | 133 | 191 | 0.91 | 0.65-1.28 |
|  | Valsartan | 20 | 57 | 77 | 1.54 | 0.81-2.90 |
|  | Candesartan | 6 | 12 | 18 | 0.91 | 0.32-2.55 |
|  | Irbesartan | 26 | 47 | 73 | 0.74 | 0.40-1.37 |
|  | Losartan | 9 | 14 | 23 | 0.68 | 0.28-1.67 |
| Angiotensin-Converting Enzyme Inhibitors | | 55 | 90 | 145 | 0.65 | 0.45-0.94 |
|  | Captopril | 7 | 21 | 28 | 2.09 | 0.82-5.30 |
|  | Fosinopril | 6 | 17 | 23 | 1.90 | 0.70-5.16 |
|  | Enalapril | 10 | 17 | 27 | 1.05 | 0.44-2.49 |
|  | Benazepril | 28 | 32 | 60 | 0.53 | 0.27-1.05 |
|  | Ramipril | 4 | 3 | 7 | 0.44 | 0.09-2.04 |
| Diuretics | | 27 | 37 | 64 | 0.56 | 0.34-0.94 |
|  | Spironolactone | 14 | 25 | 39 | 1.00 | 0.35-2.86 |
|  | Hydrochlorothiazide | 8 | 12 | 20 | 0.78 | 0.26-2.31 |
| **Antidiabetic** | | 234 | 525 | 759 | 2.43 | 2.41-3.31 |
| Sulfonylureas | | 57 | 181 | 238 | 1.63 | 1.15-2.32 |
|  | Glyburide | 1 | 9 | 10 | 2.93 | 0.36-23.64 |
|  | Repaglinide | 11 | 40 | 51 | 1.19 | 0.56-2.50 |
|  | Gliclazide | 21 | 71 | 92 | 1.11 | 0.60-2.05 |
|  | Glipizide | 6 | 20 | 26 | 1.06 | 0.40-2.77 |
|  | Glimepiride | 18 | 41 | 59 | 0.63 | 0.33-1.23 |
| Thiazolidinediones | | 16 | 30 | 46 | 0.83 | 0.45-1.56 |
|  | Rosiglitazone | 3 | 9 | 12 | 1.86 | 0.42-8.15 |
|  | Pioglitazone | 13 | 21 | 34 | 0.54 | 0.12-2.36 |
| Others | | 161 | 314 | 475 | 0.67 | 0.49-0.94 |
|  | Acarbose | 64 | 166 | 230 | 1.70 | 1.16-2.50 |
|  | Metformin | 97 | 143 | 240 | 0.55 | 0.38-0.81 |
| **Antithrombotic** | | 506 | 1091 | 1597 | 2.76 | 2.48-3.07 |
| Antiplatelet agents | | 240 | 728 | 968 | 2.22 | 1.79-2.76 |
|  | Clopidogrel | 70 | 248 | 318 | 1.25 | 0.91-1.72 |
|  | Aspirin | 170 | 480 | 650 | 0.80 | 0.58-1.10 |
| Anticoagulants | | 265 | 362 | 627 | 0.45 | 0.36-0.56 |
|  | Heparin | 130 | 221 | 351 | 1.65 | 1.20-2.27 |
|  | Dabigatran | 1 | 4 | 5 | 2.97 | 0.33-26.74 |
|  | Rivaroxaban | 9 | 19 | 28 | 1.59 | 0.71-3.57 |
|  | Warfarin | 43 | 53 | 96 | 0.89 | 0.58-1.38 |
|  | Dalteparin | 13 | 6 | 19 | 0.33 | 0.12-0.88 |
|  | Enoxaparin | 70 | 58 | 128 | 0.54 | 0.36-0.79 |
| **Antifungal** | | 643 | 936 | 1579 | 1.85 | 1.67-2.04 |
|  | Fluconazole | 214 | 415 | 629 | 1.60 | 1.30-1.97 |
|  | Voriconazole | 164 | 321 | 485 | 1.52 | 1.22-1.91 |
|  | Micafungin | 20 | 37 | 57 | 1.28 | 0.74-2.23 |
|  | Amphotericin B | 89 | 67 | 156 | 0.48 | 0.34-0.67 |
|  | Terbinafine | 23 | 16 | 39 | 0.47 | 0.25-0.89 |
|  | Ketoconazole | 21 | 13 | 34 | 0.42 | 0.21-0.84 |
|  | Itraconazole | 111 | 67 | 178 | 0.37 | 0.27-0.51 |
| **Antiulcer** | | 803 | 847 | 1650 | 1.33 | 1.20-1.46 |
| Proton Pump Inhibitors | | 708 | 794 | 1502 | 2.01 | 1.41-2.86 |
|  | Rabeprazole | 51 | 66 | 117 | 1.17 | 0.80-1.71 |
|  | Pantoprazole | 205 | 238 | 443 | 1.05 | 0.84-1.31 |
|  | Omeprazole | 325 | 359 | 684 | 0.97 | 0.79-1.19 |
|  | Lansoprazole | 127 | 131 | 258 | 0.90 | 0.69-1.18 |
| H2 Receptor Antagonists ^a^ | | 95 | 53 | 148 | 0.50 | 0.35-0.71 |
|  | Ranitidine | 30 | 22 | 52 | 1.54 | 0.77-3.09 |
|  | Cimetidine | 40 | 24 | 64 | 1.14 | 0.58-2.24 |
|  | Famotidine | 23 | 7 | 30 | 0.48 | 0.19-1.20 |
| **Antibacterial** | | 3012 | 3006 | 6018 | 1.28 | 1.21-1.35 |
| Carbapenems | | 215 | 352 | 567 | 1.73 | 1.44-2.06 |
|  | Imipenem | 60 | 124 | 184 | 1.40 | 0.97-2.03 |
|  | Meropenem | 155 | 228 | 383 | 0.71 | 0.49-1.03 |
| Fluoroquinolones | | 898 | 1177 | 2075 | 1.51 | 1.36-1.69 |
|  | Moxifloxacin | 214 | 384 | 598 | 1.55 | 1.27-1.88 |
|  | Ciprofloxacin | 23 | 30 | 53 | 1.00 | 0.57-1.73 |
|  | Norfloxacin | 4 | 1 | 5 | 0.19 | 0.02-1.70 |
|  | Levofloxacin | 657 | 762 | 1419 | 0.67 | 0.56-0.81 |
| Tetracyclines | | 56 | 62 | 118 | 1.11 | 0.77-1.60 |
|  | Minocyline | 15 | 29 | 44 | 2.40 | 1.11-5.21 |
|  | Doxycycline | 39 | 31 | 70 | 0.44 | 0.20-0.93 |
| Cephalosporins | | 637 | 564 | 1201 | 0.86 | 0.76-0.98 |
|  | Cefepime | 17 | 34 | 51 | 2.34 | 1.29-4.24 |
|  | Ceftazidime | 116 | 144 | 260 | 1.54 | 1.17-2.03 |
|  | Cefdinir | 5 | 8 | 13 | 1.82 | 0.59-5.59 |
|  | Cefprozil | 3 | 6 | 9 | 2.27 | 0.57-9.13 |
|  | Cefotaxime | 51 | 54 | 105 | 1.22 | 0.81-1.82 |
|  | Cefoperazone | 10 | 5 | 15 | 0.56 | 0.19-1.65 |
|  | Cefazolin | 33 | 23 | 56 | 0.78 | 0.45-1.34 |
|  | Ceftriaxone | 178 | 133 | 311 | 0.80 | 0.61-1.03 |
|  | Cephalexin | 5 | 1 | 6 | 0.22 | 0.03-1.93 |
|  | Cefuroxime | 186 | 147 | 333 | 0.85 | 0.66-1.10 |
|  | Cefaclor | 32 | 8 | 40 | 0.27 | 0.12-0.60 |
| Penicillins | | 165 | 111 | 276 | 0.66 | 0.52-0.85 |
|  | Penicillin | 23 | 20 | 43 | 1.36 | 0.71-2.61 |
|  | Flucloxacillin | 6 | 5 | 11 | 1.25 | 0.37-4.20 |
|  | Amoxicillin-clavulanate | 80 | 60 | 140 | 1.25 | 0.77-2.20 |
|  | Amoxicillin | 46 | 23 | 69 | 0.68 | 0.38-1.20 |
| Sulfonamides | | 151 | 98 | 249 | 0.64 | 0.49-0.83 |
|  | Sulfamethoxazole | 70 | 59 | 129 | 1.75 | 1.05-2.93 |
|  | Trimethoprim | 2 | 3 | 5 | 2.35 | 0.39-14.34 |
|  | Sulfasalazine | 77 | 35 | 112 | 0.53 | 0.32-0.90 |
| Macrolide | | 510 | 329 | 839 | 0.60 | 0.52-0.70 |
|  | Azithromycin | 393 | 280 | 673 | 1.70 | 1.18-2.46 |
|  | Clarithromycin | 57 | 41 | 98 | 1.13 | 0.74-1.74 |
|  | Erythromycin | 60 | 8 | 68 | 0.19 | 0.09-0.40 |
| Others | | 380 | 313 | 693 | 0.81 | 0.69-0.94 |
|  | Linezolid | 20 | 43 | 63 | 2.87 | 1.65-4.99 |
|  | Fosfomycin | 17 | 17 | 34 | 1.23 | 0.62-2.44 |
|  | Metronidazole | 48 | 32 | 80 | 0.79 | 0.49-1.27 |
|  | Clindamycin | 111 | 87 | 198 | 0.93 | 0.67-1.30 |
|  | Vancomycin | 182 | 130 | 312 | 0.77 | 0.57-1.05 |
| **NSAIDs** | | 418 | 303 | 721 | 0.90 | 0.78-1.05 |
| Cox-2 inhibitors | | 90 | 112 | 202 | 2.14 | 1.54-2.97 |
|  | Celecoxib | 47 | 63 | 110 | 1.18 | 0.67-2.05 |
|  | Nimesulide | 43 | 49 | 92 | 0.85 | 0.49-1.48 |
| Cox inhibitors | | 328 | 191 | 519 | 0.47 | 0.34-0.65 |
|  | Meloxicam | 25 | 30 | 55 | 2.26 | 1.28-3.97 |
|  | Diclofenac | 88 | 86 | 174 | 2.23 | 1.53-3.25 |
|  | Indomethacin | 7 | 10 | 17 | 2.53 | 0.95-6.77 |
|  | Naproxen | 6 | 4 | 10 | 1.15 | 0.32-4.12 |
|  | Ibuprofen | 107 | 31 | 138 | 0.40 | 0.26-0.63 |
|  | Acetaminophen | 89 | 26 | 115 | 0.42 | 0.26-0.68 |
| **Antineoplastic** | | 4391 | 2904 | 7295 | 0.81 | 0.77-0.85 |
| Hormonal Agents | | 20 | 89 | 109 | 6.91 | 4.24-11.25 |
|  | Flutamide | 1 | 29 | 30 | 9.18 | 1.17-71.99 |
|  | Letrozole | 5 | 13 | 18 | 0.51 | 0.16-1.65 |
| Protein Kinase Inhibitors | | 76 | 175 | 251 | 3.64 | 2.77-4.79 |
|  | Gefitinib | 12 | 80 | 92 | 4.49 | 2.26-8.91 |
|  | Bortezomib | 2 | 9 | 11 | 2.01 | 0.42-9.51 |
|  | Erlotinib | 8 | 24 | 32 | 1.35 | 0.58-3.16 |
|  | Sunitinib | 5 | 11 | 16 | 0.95 | 0.32-2.84 |
|  | Imatinib | 5 | 8 | 13 | 0.68 | 0.22-2.15 |
|  | Sorafenib | 41 | 42 | 83 | 0.27 | 0.15-0.48 |
| Alkylating Agents | | 1444 | 1299 | 2736 | 1.65 | 1.50-1.82 |
|  | Oxaliplatin | 310 | 425 | 735 | 1.78 | 1.50-2.11 |
|  | Carboplatin | 165 | 205 | 370 | 1.45 | 1.17-1.81 |
|  | Cisplatin | 361 | 366 | 727 | 1.18 | 1.00-1.39 |
|  | Temozolomide | 27 | 14 | 39 | 0.57 | 0.30-1.10 |
|  | Dacarbazine | 15 | 6 | 21 | 0.44 | 0.17-1.14 |
|  | Cyclophosphamide | 564 | 280 | 844 | 0.43 | 0.36-0.51 |
| Topoisomerase Inhibitors | | 143 | 124 | 267 | 1.33 | 1.04-1.69 |
|  | Irinotecan | 49 | 54 | 103 | 1.48 | 0.90-2.43 |
|  | Etoposide | 94 | 70 | 164 | 0.68 | 0.41-1.11 |
| Antimetabolites | | 695 | 591 | 1286 | 1.36 | 1.20-1.53 |
|  | Gemcitabine | 242 | 341 | 583 | 2.55 | 2.04-3.20 |
|  | Fluorouracil | 222 | 189 | 411 | 1.00 | 0.79-1.27 |
|  | Floxuridine | 3 | 3 | 6 | 1.18 | 0.24-5.85 |
|  | Cytarabine | 155 | 35 | 190 | 0.22 | 0.15-0.32 |
|  | Azathioprine | 69 | 23 | 92 | 0.37 | 0.23-0.60 |
| Tubulin active inhibitors | | 510 | 315 | 825 | 0.93 | 0.80-1.07 |
|  | Docetaxel | 401 | 276 | 677 | 1.92 | 1.29-2.86 |
|  | Vincristine | 109 | 39 | 148 | 0.52 | 0.35-0.77 |
| Antibiotics | | 114 | 41 | 155 | 0.54 | 0.37-0.77 |
|  | Doxorubicin | 85 | 32 | 117 | 1.21 | 0.52-2.84 |
|  | Dactinomycin | 23 | 1 | 24 | 0.10 | 0.01-0.76 |
|  | Mitoxantrone | 6 | 2 | 8 | 0.92 | 0.18-4.77 |
| Others | | 1389 | 270 | 1659 | 0.22 | 0.19-0.25 |
|  | Trastuzumab | 27 | 24 | 51 | 4.92 | 2.79-8.67 |
|  | Hydroxyurea | 5 | 6 | 11 | 6.29 | 1.91-20.76 |
|  | Lenalidomide | 36 | 30 | 66 | 4.70 | 2.84-7.77 |
|  | Asparaginase | 40 | 13 | 53 | 1.71 | 0.90-3.23 |
|  | Methotrexate | 1281 | 197 | 1478 | 0.23 | 0.16-0.32 |
| **Antidepressant** | | 816 | 489 | 1305 | 0.74 | 0.66-0.83 |
| Tricyclics | | 30 | 22 | 52 | 1.23 | 0.70-2.17 |
|  | Amitriptyline | 6 | 8 | 14 | 2.29 | 0.66-7.95 |
|  | Doxepin | 16 | 12 | 28 | 1.05 | 0.35-3.17 |
|  | Clomipramine | 8 | 2 | 10 | 0.28 | 0.05-1.45 |
| Others | | 145 | 110 | 247 | 1.34 | 1.02-1.77 |
|  | Trazodone | 11 | 12 | 23 | 1.49 | 0.63-3.52 |
|  | Mirtazapine | 131 | 93 | 224 | 0.58 | 0.27-1.24 |
|  | Nefazodone | 1 | 4 | 5 | 5.43 | 0.60-49.32 |
| SNRI ^b^ | | 154 | 100 | 254 | 1.11 | 0.83-1.46 |
|  | Duloxetine | 55 | 46 | 101 | 1.53 | 0.92-2.56 |
|  | Venlafaxine | 99 | 54 | 153 | 0.65 | 0.39-1.09 |
| SSRI ^c^ | | 487 | 257 | 744 | 0.75 | 0.60-0.94 |
|  | Paroxetine | 143 | 84 | 227 | 1.17 | 0.84-1.62 |
|  | Sertraline | 143 | 77 | 220 | 1.03 | 0.74-1.43 |
|  | Citalopram | 171 | 81 | 252 | 0.85 | 0.62-1.17 |
|  | Fluoxetine | 30 | 15 | 45 | 0.94 | 0.50-1.79 |
| **Antitubercular** | | 13255 | 7983 | 21238 | 0.66 | 0.64-0.68 |
|  | Ethambutol | 2755 | 1783 | 4583 | 1.10 | 1.02-1.17 |
|  | Rifabutin | 17 | 13 | 30 | 1.27 | 0.62-2.62 |
|  | Isoniazid | 3314 | 2082 | 5396 | 1.06 | 1.00-1.13 |
|  | Protionamide | 98 | 59 | 157 | 1.00 | 0.72-1.38 |
|  | Rifampin | 3825 | 2197 | 6022 | 0.94 | 0.88-1.00 |
|  | Pyrazinamide | 3246 | 1849 | 5095 | 0.93 | 0.87-0.99 |
| **Antirheumatic** | | 884 | 459 | 1343 | 0.64 | 0.57-0.72 |
|  | Leflunomide | 168 | 171 | 339 | 2.53 | 1.96-3.26 |
|  | Hydroxychloroquine | 40 | 27 | 67 | 1.32 | 0.80-2.18 |
|  | Cyclosporine | 117 | 64 | 181 | 1.06 | 0.77-1.47 |
|  | Tocilizumab | 20 | 10 | 30 | 0.96 | 0.45-2.07 |
|  | Infliximab | 6 | 2 | 8 | 0.64 | 0.13-3.19 |
|  | Methylprednisolone | 335 | 149 | 484 | 0.79 | 0.62-1.00 |
|  | Tacrolimus | 88 | 23 | 111 | 0.48 | 0.30-0.77 |
|  | Mycophenolate | 72 | 10 | 82 | 0.25 | 0.13-0.49 |
|  | Adalimumab | 32 | 1 | 33 | 0.06 | 0.01-0.43 |
| **Anticonvulsants** | | 1254 | 545 | 1799 | 0.53 | 0.48-0.59 |
|  | Carbamazepine | 116 | 99 | 215 | 2.18 | 1.63-2.91 |
|  | Phenytoin | 32 | 23 | 55 | 1.68 | 0.98-2.90 |
|  | Gabapentin | 13 | 9 | 22 | 1.60 | 0.68-3.77 |
|  | Topiramate | 8 | 6 | 14 | 1.73 | 0.60-5.02 |
|  | Phenobarbital | 54 | 25 | 79 | 1.07 | 0.66-1.74 |
|  | Oxcarbazepine | 53 | 21 | 74 | 0.91 | 0.54-1.52 |
|  | Clonazepam | 99 | 31 | 130 | 0.70 | 0.46-1.07 |
|  | Valproate | 838 | 325 | 1163 | 0.73 | 0.60-0.90 |
|  | Lamotrigine | 25 | 3 | 28 | 0.27 | 0.08-0.91 |
| **Antiviral** | | 207 | 79 | 286 | 0.48 | 0.37-0.62 |
|  | Ritonavir | 4 | 4 | 8 | 2.71 | 0.66-11.10 |
|  | Nevirapine | 69 | 28 | 97 | 1.10 | 0.64-1.89 |
|  | Zidovudine | 34 | 14 | 48 | 1.10 | 0.55-2.17 |
|  | Efavirenz | 91 | 30 | 121 | 0.78 | 0.46-1.33 |
|  | Lopinavir | 7 | 1 | 8 | 0.37 | 0.04-3.03 |
| **Antipsychotic** | | 5283 | 1046 | 6329 | 0.22 | 0.20-0.23 |
| First generation | | 394 | 107 | 501 | 1.41 | 1.13-1.77 |
|  | Chlorpromazine | 194 | 67 | 261 | 1.73 | 1.11-2.68 |
|  | Prochlorperazine | 102 | 33 | 135 | 1.28 | 0.80-2.04 |
|  | Haloperidol | 98 | 7 | 105 | 0.21 | 0.10-0.47 |
| Second generation | | 4889 | 939 | 5828 | 0.71 | 0.56-0.89 |
|  | Olanzapine | 1487 | 378 | 1865 | 1.54 | 1.33-1.78 |
|  | Quetiapine | 852 | 210 | 1062 | 1.36 | 1.15-1.62 |
|  | Ziprasidone | 110 | 12 | 122 | 0.56 | 0.31-1.02 |
|  | Clozapine | 954 | 137 | 1091 | 0.70 | 0.58-0.86 |
|  | Risperidone | 1486 | 202 | 1688 | 0.63 | 0.53-0.74 |

*Note.* ^a^ H2 Receptor Antagonists indicated histamine type-2 receptor antagonists. ^b^ SNRI indicated serotonin and norepinephrine reuptake inhibitor; ^c^ SSRI indicated selective serotonin reuptake inhibitor.

Drugs with reporting frequency (RF) of less than 5 did not appear in the list.

**Supplementary Table 2 RR for different age groups**

| **Age groups** | **Cases** | **Number of people nationwide (×10^4^)** | **RR** | |
| --- | --- | --- | --- | --- |
|  |  |  | **Ratio** | **95% CI** |
| 0 - 9 | 1291 | 15507.2 | 0.16 | 0.15-0.17 |
| 10 - 19 | 2764 | 14687.5 | 0.37 | 0.36-0.39 |
| 20 - 29 | 9573 | 22791.3 | 0.87 | 0.85-0.89 |
| 30 - 39 | 8113 | 19868.6 | 0.84 | 0.82-0.86 |
| 40 - 49 | 11431 | 24118.4 | 1.00 | 0.98-1.02 |
| 50 - 59 | 12319 | 17918.3 | 1.56 | 1.53-1.59 |
| 60 - 69 | 10513 | 13087.1 | 1.83 | 1.79-1.88 |
| 70 - 79 | 5779 | 6234.6 | 2.05 | 1.99-2.11 |
| 80 - 89 | 2549 | 2327.5 | 2.37 | 2.27-2.48 |
| ≥ 90 | 370 | 257.7 | 3.05 | 2.71-3.42 |
